# Lymphocyte apoptosis in children with central nervous system tuberculosis: a case control study

**DOI:** 10.1186/1471-2431-11-108

**Published:** 2011-11-23

**Authors:** Paola Di Carlo, Alessandra Casuccio, Amelia Romano, Daria Spicola, Lucina Titone, Nadia Caccamo, Francesco Dieli, Caterina Mammina, Elisabetta Pace, Mark Gjomarkaj, Mario Melis, Manlio Tolomeo

**Affiliations:** 1 Department of Sciences for Health Promotion, University of Palermo, Via del Vespro 133, Palermo I-90127, Italy; 2 Department of Experimental Biomedicine and Clinical Neuroscience, University of Palermo, Via del Vespro 133, Palermo I-90127, Italy; 3 Pediatric Infectious Diseases, G. Di Cristina Children's Hospital, ARNAS Civico, P.zza Montalto, 8, Palermo I-90157, Italy; 4 Department of Biopathology, University of Palermo, Corso Tukory 211, Palermo I- 90134, Italy; 5 CNR Institute of Biomedicine and Molecular Immunology, Via Ugo La Malfa 153, Palermo I-90146, Italy

## Abstract

**Background:**

Studies of the apoptosis mechanisms involved in the pathogenesis of tuberculosis have suggested that *Mycobacterium tuberculosis *can actively interfere with the apoptosis of infected cells. *In vivo *studies have been performed in adult populations but have not focused on this process in children. In the present study, we analyzed spontaneous T lymphocyte (PBT) apoptosis in the peripheral blood of children with central nervous system tuberculosis (CNS TB), before and after chemotherapy, and compared the results with healthy controls.

**Methods:**

A case-control study was conducted from January 2002 to June 2009. It included 18 children with CNS TB and 17 healthy controls. Spontaneous apoptosis of PBTs, including CD4^+^, CD8^+ ^and CD8^+^/CD28^+ ^T cells, was evaluated after 24 and 72 h of culture in complete medium, using the Annexin V detection test. Analysis was conducted before and after chemotherapy, and expression of the apoptotic markers CD95 (Fas) and Fas ligand (FasL) was evaluated.

**Results:**

Higher percentages of apoptotic T cells and CD4 lymphocytes were isolated from children with acute phase CNS TB than from children in the control group (p < 0.05). This difference significantly decreased after 60 days of specific treatment. In children with CNS TB, high levels of Fas ligand expression were detected in lymphocyte populations, associated with a high percentage of Fas positive cells, before and after treatment. In contrast to the CD4+ apoptosis profile, we did not find any significant difference in total CD8^+ ^cell apoptosis between children with acute phase disease and the control group. However, the percentage of apoptotic CD8^+^/CD28^+ ^T cells was significantly higher in the children with acute phase disease than in the healthy controls.

**Conclusions:**

Our findings indicate that CNS TB in pediatric patients increases the sensitivity of CD4 and CD8^+^/CD28^+ ^T cells to apoptosis, suggesting a hypoergic status of this infection. This could play a key role in the immunopathogenesis of this complicated form of TB. Interestingly, specific chemotherapy is able to normalize both apoptosis sensitivity and T-cell activation.

## Background

Pediatric tuberculosis (TB) remains one of the most important communicable diseases and represents a major health problem in the developing world. Globally, approximately 1 million new cases and approximately 500,000 deaths due to pediatric TB are reported every year [1,2].

In Italy, similar to many other industrialized nations, TB is a relatively rare disease. In the last few years, the incidence of TB has been less than 10 cases/100,000 population (in 2009 the rate was 7/100,000 population, corresponding to just over 4,200 cases) while according to WHO estimates, the prevalence of latent infections is 12% (corresponding to just over 7,200,000 latent infections) [1].

While 90% of immunocompetent adult patients infected by the tubercle bacillus will not develop the disease during their lifetime, children have shown various clinical forms of infection and progressive disease [2,3]. Although pulmonary TB tends to be the most common form of TB, TB of the central nervous system (CNS) carries the highest mortality and morbidity rates and develops in 4% of infected children, especially during infancy [4-6].

It is well known that the immune system of a child is too immature to stimulate a host protective response against intracellular pathogen growth. Some progress towards understanding the mechanism involved in immune responses to *Mycobacterium tuberculosis *infection has been made by studying adult patients. However, other aspects need to be assessed through research in pediatric populations. The relevant role of apoptosis (programmed cell death) in the development of the immune system in children has recently been highlighted in studies on the efficacy of the TB vaccine [7,8].

Recent studies have demonstrated that *M. tuberculosis *actively interferes with the apoptosis of infected cells *in vitro *as a means of virulence, and that dysregulation of the host's lipid metabolism is a major pathway for generating pathology and promoting necrosis over apoptosis [9,10]. However, the importance of apoptosis as a virulence mechanism *in vivo *and interaction of apoptotic mechanisms with the host cytokine response have been largely unexplored until recently, with this area of research slowly being understood [10-12].

A number of alternative mechanisms of peripheral T cell apoptosis have been defined. The distinction has been made between active apoptosis, which follows stimulation and activation of the T cells, and passive apoptosis, which occurs as a result of withdrawal of the sustaining cytokines following activation [13-16]. Activation of death receptors by their ligands can initiate apoptosis. Fas and Tumor Necrosis Factor (TNF) receptors are more clearly understood than others, and their important role in peripheral T cell apoptosis has been demonstrated. The Fas/Fas ligand (FasL) interaction is particularly important in initiating activation induced cell death of CD4^+ ^T cells [17-20].

Understanding the specific mechanisms by which children's T cells fail to contain Mycobacterium TB infection could offer potential new targets for appropriate vaccines. In addition, studying the defects in infant immune responses that explain poor bacterial control may contribute more broadly to our understanding of severe forms of TB such as CNS TB.

In this study, we analyzed spontaneous T lymphocyte (PBT) apoptosis and the subsets CD3+, CD4+ and CD8+, including the analysis of apoptotic CD8^+^/CD28^+ ^T cells, in the peripheral blood of children with CNS TB, before and after starting anti tubercular treatment and compared them with healthy control children.

## Methods

### Subjects

Eighteen children with CNS TB (10 boys, 8 girls; mean age [± SD], 3.8 years [3.4]; range, 0.5-12 years] admitted to the G. Di Cristina Children's Hospital in Palermo, Italy, were selected according to Centers for Disease Control (CDC) Case Definition clinical and/or microbiological criteria between January 2002 and December 2009 [21].

Diagnosis was bacteriologically confirmed by polymerase chain reaction (PCR) and/or culture of *M. tuberculosis *complex in CSF samples and/or CSF culture, as previously reported [22,23]. CSF culture and PCR were positive in 60% and 90% of patients, respectively. Resistant strains to 1 or more anti-TB drugs were found in 4 children and their adult sources.

Seven patients (38%) had pulmonary TB. The source of infection was documented for 9 patients (50%): it was grandparents for 4 patients, parents for 3 patients, a teacher for 1 patient, and a neighbor for 1 patient.

All of the children received antitubercular treatment for 1 year, consisting of 2 months of isoniazid (INH), rifampicin (RMP), pyrazinamide (PZA) and streptomycin (SM) or ethambutol (ETB), followed by 10 months of RMP and INH [24].

All the subjects enrolled in the study were negative for human immunodeficiency virus (HIV).

A control group comprised 17 healthy children who were Purified Protein Derivative (PPD) skin test negative (10 boys and 7 girls; mean age [± SD], 5.6 years [3.2]; range, 2-12 years). The study protocol was approved by the Ethics Committee of the G. Di Cristina Hospital in Palermo (Italy) and informed consent was sought in accordance with the principles of the Declaration of Helsinki. Patients were thoroughly informed about the procedures and written informed consent was obtained from each of them. Peripheral blood was drawn from children with CNS TB during the acute phase of illness, and 30-60 days and 90 days after starting chemotherapy. The follow-up time point after the start of antitubercular therapy was chosen on the basis of previous studies conducted by our group [22,25,26].

### Isolation of peripheral blood mononuclear cells (PBMCs)

PBMCs were isolated by Ficoll-Paque density centrifugation from 20 mL of heparinized blood drawn from children with CNS-TB and from the healthy children in the control group.

PBMC cultures: PBMCs were cultured in RPMI 1640 medium supplemented with 10% FCS at 37°C in a humidified atmosphere of 5% CO_2 _[27,28].

### Apoptosis detection and PBT phenotype analysis

T cell spontaneous apoptosis was determined after 24 and 72 h of culture in complete medium, using the Annexin V detection kit as previously described [27,28]. Apoptotic cells were identified by means of double or triple staining with an Annexin V-FITC kit (Bender MedSystem, Vienna, Austria) and anti-human peridinin chlorophyll protein-CD3 (Becton Dickinson, San Jose, CA, USA), and anti-human phycoerythrin (RPE)-conjugated CD8 (Dakopatts, Glostrup, Denmark) or anti-human RPE-conjugated CD4 clone MT310 (Dakopatts). T lymphocytes were also investigated for their expression of CD28 (Dakopatts). Cell apoptosis and the phenotype of PBTs were determined with a FACSCalibur Plus flow cytometer (Becton Dickinson). Phycoerythrin-conjugated (X-928, Dakopatts) and FITC-conjugated mouse anti-human IgG1 (X-927, Dakopatts) were used as negative controls.

### Fas and Fas-L expression

Fas and FasL expression were evaluated by flow cytometry (Becton Dickinson) using antihuman PE-conjugated CD95 and antihuman FITC conjugated CD95L (Dakopatts) MoAbs. PE-conjugated (X-928, Dakopatts) and FITC-conjugated mouse antihuman IgG1 (X-927, Dakopatts) were used as negative controls. Fas and FasL expressions are shown as the percentage of positive cells.

### Statistical analysis

Discrete variables were analyzed using the chi-square and Fisher's exact tests, as needed. The independent Student's t test and the Mann-Whitney U statistic test were used for parametric and non-parametric analysis, respectively. The paired-samples Student's t-test and paired Wilcoxon signed-rank test were used for intragroup parametric and non-parametric analysis, respectively. All statistical tests were two-tailed and were applied at the 5% significance level. Data were analyzed with Epi Info software, version 3.2.2 (Centers for Disease Control and Prevention, Atlanta, Georgia, USA) and SPSS software (version 14.0; SPSS Inc., Chicago, IL, USA).

## Results

### T lymphocytes, Fas and FasL during CNS TB

The mean (± SD) total lymphocyte and CD3^+ ^lymphocyte counts in peripheral blood of patients in the acute phase of disease and in controls are shown in Table 1. There were no significant differences between the 2 groups (p > 0.05).

**Table 1:** Peripheral blood lymphocyte populations* in acute CNS TB children and uninfected children (controls)

	Acute phase	Controls	P-value
Lymphocytes (× 10^9^/l)	6.77(3.6)	6.58(2.8)	0.863
CD3+ T -lymphocytes (× 10^9^/l)	6.20(3.2)	5.11(2.1)	0.245
CD4+ T -lymphocytes (× 10^9^/l)	2.17(1.2)	2.28(0.8)	0.753
CD8+ T -lymphocytes (× 10^9^/l)	1.33(0.6)	1.09(0.4)	0.176
CD28+ T -lymphocytes (× 10^9^/l)	0.36(0.2)	0.27(0.1)	0.105

* mean values (± SD)

Spontaneous apoptosis of PBTs isolated from CNS TB patients and from the healthy controls was evident after 24 h (p < 0.0005), and steadily increased after 72 h (p < 0.0005).

**Figure 1:**
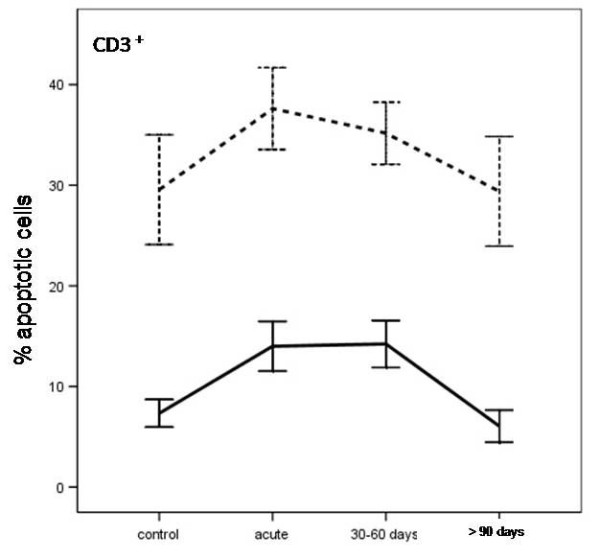
**Apoptosis in T-lymphocytes from uninfected children (controls), and children with CNS tuberculosis before and after specific treatment, at the different time periods**. Apoptosis was induced *in vitro *within 24 h (solid line) and after 72 h of culture in complete medium (dashed line).

There was a significant increase in apoptotic CD3^+ ^lymphocytes in the children with acute phase CNS TB after 24 (p < 0.0005) and 72 h of incubation compared with those in the control group (p < 0.0005). This increased sensitivity in CD3^+ ^lymphocytes to apoptosis was still evident after 30-60 days of specific anti-tubercular therapy, but it was decreased in children treated for more than 90 days (Figure 1).

**Figure 2:**
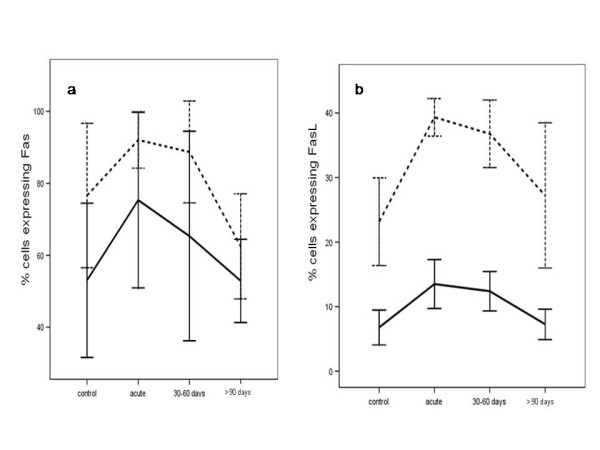
**Fas (2a) and FasL (2b) expression during central nervous system tuberculosis in the illness group, before and after specific treatment at the different time periods, and in uninfected children (controls)**. Results are shown as percentage of Fas and FasL -expressing cells among T-lymphocytes after 24 h (solid line) or within 72 h of culture in complete medium (dashed line).

Fas and FasL expression was significantly higher in the T lymphocytes of children with acute phase CNS TB than that in healthy control group children after 24 and 72 h of incubation (p < 0.0005, Figure 2a, b). This difference was still evident in patients treated for 30-60 days (Fas and FasL after 24 h, p = 0.010, p < 0.0005; Fas and FasL after 72 h, p < 0.0005, p < 0.0005), but it was decreased in children treated for more than 90 days (Fas and FasL after 24 h of incubation, p > 0.05, p = 0.024; Fas and FasL after 72 h of incubation, p = 0.003, p = 0.025; Figure 2a, b).

### T Lymphocyte subset apoptosis during CNS TB

The mean (± SD) CD4^+^, CD8^+ ^and CD8^+^/CD28^+ ^T lymphocyte counts in patients during the acute phase of the disease and in the controls are shown in Table 1. There were no significant differences between the 2 groups (p > 0.05).

**Figure 3:**
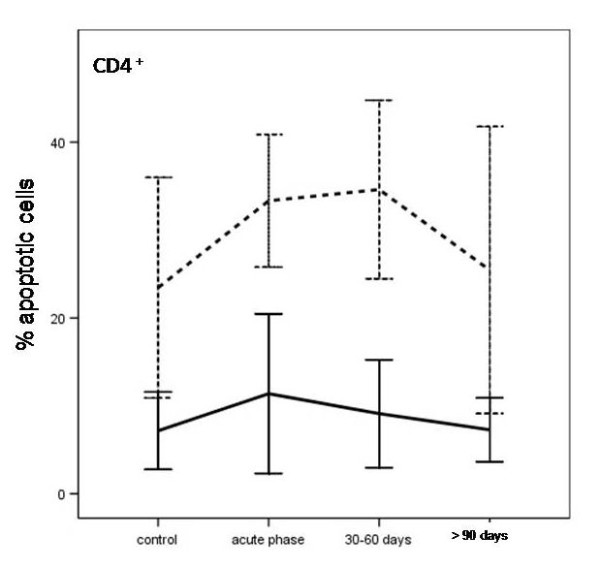
**Apoptosis in CD4^+ ^T cells from children with CNS tuberculosis, before and after specific treatment at the different time periods, and from uninfected children (controls)**. Results are shown as percentage of CD4^+ ^T cells after 24 h (solid line) or within 72 h of culture in complete medium (dashed line).

Spontaneous apoptosis was evaluated in different CD3^+ ^lymphocyte subpopulations (CD4^+^, CD8^+^) of patients affected by CNS TB and in children in the control group. As shown in Figure 3, only CD4^+ ^T-lymphocytes isolated from the TB children before treatment showed a greater sensitivity towards apoptosis than those of the control group (acute vs control after 24 and 72 h of incubation, p = 0.019 and p < 0.0005, respectively). This difference was decreased after 30-60 days of antitubercular treatment (after 24 and 72 h of incubation, p = 0.027 and p = 0.035, respectively) and disappeared after 90 days (acute vs control after 24 and 72 h of incubation, p > 0.05).

Comparative analysis of apoptosis in CD8^+ ^lymphocyte populations in acute phase CNS TB children treated for 30-60 days and for more than 90 days and in the controls showed no significant differences after 24 and 72 h of incubation (p > 0.05).

**Figure 4:**
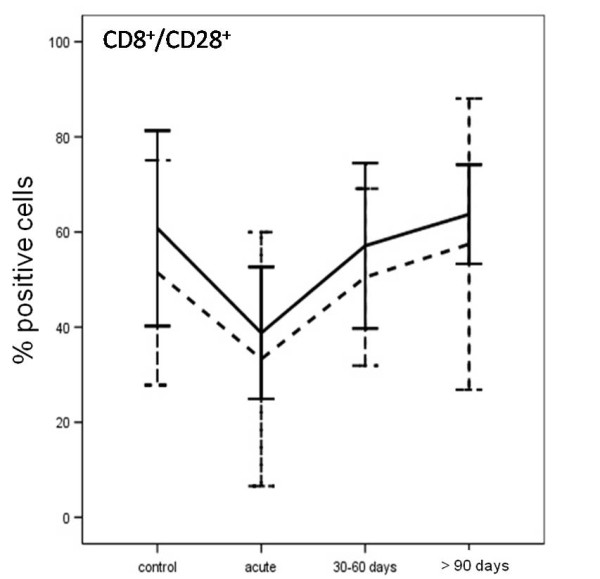
**Percentage of CD8^+^/CD28^+ ^T from children with CNS tuberculosis, before and after specific treatment at the different time periods, and from uninfected children (controls)**. Results are shown as percentage of CD8^+^/CD28^+ ^T cells after 24 h (solid line) or within 72 h of culture in complete medium (dashed line).

Analysis of apoptotic CD8^+^/CD28^+ ^T cells showed impaired cell apoptosis in the acute phase of disease (acute vs control after 24 and 72 h of incubation, p < 0.0005 and p = 0.002, respectively). The difference between CNS TB patients and healthy control children decreased after 90 days of specific treatment (p > 0.05, Figure 4).

## Discussion

The results of our study show that PBTs isolated from children with CNS TB had a stronger apoptotic response and underwent apoptosis more rapidly than those of healthy subjects, even after 3 months of antitubercular treatment. These responses were due to high levels of FaL expression in lymphocyte populations. Moreover, the number of Fas positive cells remained high even after starting treatment for TB. The reason for these findings may be because Koch's bacillus produces or induces substances in macrophages that increase the sensitivity of lymphocytes to apoptosis by increasing Fas expression or Fas-L for protection against antitubercular activity [9,29].

We did not find any significant difference in the absolute number of T lymphocytes and their subsets in the peripheral blood in children with CNS TB and healthy controls. A previous study of adult pulmonary TB patients showed a significantly lower percentage of CD3^+ ^and CD4^+ ^cells, and a significantly lower ratio of CD4^+ ^and CD8^+ ^cells compared with healthy controls [30].

CD4^+ ^cells are critical in immunity against *M. tuberculosis *infection. CD4+ T lymphocytes play 2 important roles in TB infection: first, they produce cytokines that govern cell-mediated immune responses; and second, they eliminate infected macrophages via apoptosis [31-33].

The relevant role of CD4^+ ^cells in the pathogenesis of CNS TB is confirmed by CD4^+ ^cell counts and TB meningitis in the HIV population [33]. We observed a high percentage of CD4^+ ^T lymphocyte apoptosis in our study population, both in the acute phase of disease and after the start of specific treatment. Therefore, CD4 depletion by apoptosis is a foreseeable phenomenon in TB patients. In fact, we found that patients with a more severe form of TB had CD4^+ ^lymphocyte depletion despite their CD4^+ ^cell counts. In contrast to the CD4^+ ^apoptosis profile, we did not find any significant difference in CD8^+ ^cell apoptosis between children with CNS TB and the control group. This could be the consequence of increased CD4^+ ^lymphocyte apoptosis through the Fas/FasL system.

CD8^+ ^T cells can efficiently eradicate TB infection by inducing apoptosis of infected cells, and this cell death is mainly, but not always, mediated via the Tumor Necrosis Factor Receptors (TNFR) [17,34,35]. The hypoergic status of CD8 T cells and the increased susceptibility of CD4 T cells to apoptosis may be induced by specific cytokines produced during infection to avoid an immunological response against Koch's bacillus [14,35]. Although different *M. tuberculosis *specific CD8^+^T cell subsets, such as CD27^+ ^and CD28^+ ^(activated T cells), may be involved in these mechanisms, their role is not well understood in pediatric patients [31,36].

Interestingly, CD8^+^/CD28^+ ^T cell apoptosis was significantly lower in the children with CNS TB before chemotherapy, suggesting a protective role of these cells in response to *M. tuberculosis *infection. Contrasting results have been reported in other studies. Both increases and decreases in levels of CD8^+^/CD28^+ ^T cells in peripheral blood have been detected in adult patients with active TB compared with healthy controls [36,37]. However, further studies are required to elucidate the mechanisms of these phenomena.

Our study suggests that TB infection in children increases the sensitivity of CD4^+ ^T lymphocytes to apoptosis and causes a decrease in CD8^+ ^activated T lymphocytes. Interestingly, chemotherapy was able to normalize both apoptosis sensitivity and T cell activation. This could play a key role in the immune pathogenesis of the disease. Involvement of the CNS is a serious complication of TB and is most frequent in children aged between 6 months and 6 years, although it can occur at any age [4,5]. Ten of the children in our study population were in this age range. Consequently, our data may help the understanding of what type of immune response against the tubercle bacillus is activated in the first year of life.

## Conclusions

To our knowledge, this is the only study to date to evaluate apoptosis in T lymphocytes from children with CNS TB. A better understanding of apoptosis regulation in TB is required to develop new immune-based therapies aimed at modulating apoptosis in infected patients. Considering the current, very poor laboratory support for the diagnosis and management of childhood TB, further studies with large numbers of subjects are required to define the clinical value of these assays.

## List of abbreviations

FACS: Fluorescence Activated Cell Sorting; CD: Cluster differentiation; FITC: fluorescein- isothiocyanate; PE: phycoerythrin.

## Competing interests

The authors declare that they have no competing interests.

## Authors' contributions

PD designed the study and drafted the manuscript. NC, FD, BP, DS and MG set up and conducted the study in the field and contributed to the interpretations of results. MM and MT were responsible for laboratory analysis. AC was responsible for the acquisition, statistical analysis and interpretation of data. The ethics application and funding applications were submitted by PD, AR and LT as the principal pediatric investigators. MC provided microbiological data. MT supported apoptosis laboratory results, supervised the study and revised the manuscript. All authors have read and approved the final manuscript.

## Pre-publication history

The pre-publication history for this paper can be accessed here:

http://www.biomedcentral.com/1471-2431/11/108/prepub

## References

[B1] WHO Global tuberculosis control 2010 WHO Report 2010

[B2] UNICEF The state of the World's children Unicef Main Report 2011

[B3] Newton SM Brent AJ Anderson S Whittaker E Kampmann B Paediatric tuberculosis Lancet Infect Dis 2008 8 498 510 10.1016/S1473-3099(08)70182-8 18652996 PMC2804291

[B4] Swaminathan S Rekha B Pediatric tuberculosis: global overview and challenges Clin Infect Dis 2010 50 184 194 10.1086/649209 20397947

[B5] Garg RK Tuberculosis of the central nervous system Postgrad Med J 1999 75 133 140 10448488 10.1136/pgmj.75.881.133 PMC1741157

[B6] Be NA Kim KS Bishai WR Jain SK Pathogenesis of central nervous system tuberculosis Curr Mol Med 2009 9 94 99 10.2174/156652409787581655 19275620 PMC4486069

[B7] Hanekom WA The immune response to BCG vaccination of newborns Ann N Y Acad Sci 2005 1062 69 78 10.1196/annals.1358.010 16461790

[B8] Soares AP Scriba TJ Joseph S Harbacheuski R Murray RA Gelderbloem SJ Hawkridge A Hussey GD Maecker H Kaplan G Hanekom WA Bacillus Calmette-Guérin vaccination of human newborns induces T cells with complex cytokine and phenotypic profiles J Immunol 2008 1 3569 3577 10.4049/jimmunol.180.5.3569 PMC2842001 18292584

[B9] Mustafa T Bjune TG Jonsson R Pando RH Nilsen R Increased expression of fas ligand in human tuberculosis and leprosy lesions: a potential novel mechanism of immune evasion in mycobacterial infection Scand J Immunol 2001 54 630 639 10.1046/j.1365-3083.2001.01020.x 11902340

[B10] Briken V Miller JL Living on the edge: inhibition of host cell apoptosis by Mycobacterium tuberculosis Future Microbiol 2008 3 415 422 10.2217/17460913.3.4.415 18651813 PMC2650273

[B11] Abebe M Kim L Rook G Aseffa A Wassie L Zewdie M Zumla A Engers H Andersen P Doherty TM Modulation of cell death by M. tuberculosis as a strategy for pathogen survival Clin Dev Immunol 2011 2011 678570 Epub 2011 Jan 4 21253484 10.1155/2011/678570 PMC3022200

[B12] Behar SM Divangahi M Remold HG Evasion of innate immunity by mycobacterium tuberculosis: is death an exit strategy? Nat Rev Microbiol 2010 8 668 674 20676146 10.1038/nrmicro2387 PMC3221965

[B13] Boom WH Wallis RS Chervenak KA Human Mycobacterium tuberculosis-reactiveCD4+ T-cell clones: heterogeneity in antigen recognition, cytokine production, and cytotoxicity for mononuclear phagocytes Infect Immun 1991 59 2737 2743 1713198 10.1128/iai.59.8.2737-2743.1991 PMC258080

[B14] Dubaniewicz A Trzonkowski P Dubaniewicz-Wybieralska M Dubaniewicz A Singh M Myśliwski A Mycobacterial heat shock protein-induced blood T lymphocytes subsets and cytokine pattern: comparison of sarcoidosis with tuberculosis and healthy controls Respirology 2007 12 346 354 10.1111/j.1440-1843.2007.01076.x 17539837

[B15] Arcila ML Sánchez MD Ortiz B Barrera LF García GAS Rojas M Activation of apoptosis, but not necrosis, during Mycobacterium tuberculosis infection correlated with decreased bacterial growth: role of TNF-alpha, IL-10, caspases and phospholipase A2 Cell Immunol 2007 249 80 93 10.1016/j.cellimm.2007.11.006 18160064

[B16] Cowley SC Elkins KL CD4+ T cells mediate IFN-gamma-independent control of Mycobacterium tuberculosis infection both in vitro and in vivo J Immunol 2003 171 4689 4699 14568944 10.4049/jimmunol.171.9.4689

[B17] Sytwu HK Liblau RS McDevitt HO The roles of Fas/APO-1 (CD95) and TNF in antigen-induced programmed cell death in T cell receptor transgenic mice Immunity 1996 5 17 30 10.1016/S1074-7613(00)80306-4 8758891

[B18] Wong B Choi Y Pathways leading to cell death in T cells Curr Opin Immunol 1997 9 358 364 10.1016/S0952-7915(97)80082-9 9203409

[B19] Mrugacz M Fas expression in conjunctival epithelial cells of patients with cystic fibrosis J Interferon Cytokine Res 2009 29 735 740 10.1089/jir.2008.0111 19642903

[B20] Zhong J Gilbertson B Cheers C Apoptosis of CD4+ and CD8+ T cells during experimental infection with Mycobacterium avium is controlled by Fas/FasL and Bcl-2-sensitive pathways, respectively Immunol Cell Biol 2003 81 480 486 10.1046/j.1440-1711.2003.01193.x 14636245

[B21] CDC Case definitions for infectious conditions under public health surveillance MMWR 1997 46 40 41 9148133

[B22] Nastasi A Mammina C Epidemiological study of tuberculosis in Palermo, Italy: IS6110 fingerprinting of Mycobacterium tuberculosis strains isolated in the years 1994-1998 Infection 1999 27 318 222 10.1007/s150100050036 10624590

[B23] Saporito L Florena AM Colomba C Pampinella D Di Carlo P Prurigo nodularis due to Mycobacterium tuberculosis J Med Microbiol 2009 58 1649 1651 10.1099/jmm.0.007518-0 19661207

[B24] American Thoracic Society Treatment of tuberculosis and tuberculosis infection in adults and children Am J Respir Crit Care Med 1994 149 1359 1374 8173779 10.1164/ajrccm.149.5.8173779

[B25] Di Carlo P Romano A Melis M Abbagnato L Caccamo N Meraviglia S Dieli F An overview of the role of T cells in controlling tuberculosis infection in a pediatric population J Ped Infect Dis 2009 4 221 228

[B26] Melis MR Pace E Di Carlo P Romano A Titone L Vignola AM Ferraro M Tipa A Bonsignore G Gjomarkai M Increased peripheral T-Lymphocyte apoptosis in Childhood tuberculosis Am J Respir Crit Care Med 2002 165 289 International Conference, Atlanta, Georgia. American Thoracic Society

[B27] Pace E Gjomarkaj M Melis M Profita M Spatafora M Vignola AM Bonsignore G Mody CH Interleukin-8 induces lymphocyte chemotaxis into the pleural space. Role of pleural macrophages Am J Respir Crit Care Med 1999 159 1592 1599 10228132 10.1164/ajrccm.159.5.9806001

[B28] Tolomeo M Di Carlo P Abbadessa V Titone L Miceli S Barbusca E Cannizzo G Mancuso S Arista S Scarlata F Monocyte and Lymphocyte apoptosis resistance in acute and chronic brucellosis and its possible implications in Clinical Management Clin Infect Dis 2003 36 1533 1538 10.1086/375223 12802752

[B29] Gallegos AM van Heijst JW Samstein M Su X Pamer EG Glickman MS A gamma interferon independent mechanism of CD4 T cell mediated control of M. tuberculosis infection in vivo PLoS Pathog 2011 7 e1002052 10.1371/journal.ppat.1002052 21625591 PMC3098235

[B30] Wu YE Zhang SW Peng WG Li KS Li K Jiang JK Lin JH Changes in lymphocyte subsets in the peripheral blood of patients with active pulmonary tuberculosis J Int Med Res 2009 37 1742 1749 20146872 10.1177/147323000903700610

[B31] Kapina MA Shepelkova GS Mischenko VV Sayles P Bogacheva P Winslow G Apt AS Lyadova IV CD27low CD4 T lymphocytes that accumulate in the mouse lungs during mycobacterial infection differentiate from CD27high precursors in situ, produce IFN-gamma, and protect the host against tuberculosis infection J Immunol 2007 178 976 985 17202360 10.4049/jimmunol.178.2.976

[B32] Winslow GM Cooper A Reiley W Chatterjee M Woodland DL Early T-cell responses in tuberculosis immunity Immunol Rev 2008 225 284 299 10.1111/j.1600-065X.2008.00693.x 18837789 PMC3827678

[B33] Vidal JE de Oliveira AC Hernández AV CD4+ T-cell count and cerebrospinal fluid findings in HIV-infected patients with tuberculous meningitis Int J Tuberc Lung Dis 2010 14 1496 1497 20937194

[B34] Alexander-Miller MA Leggatt GR Sarin A Berzofsky JA Role of antigen, CD8, and cytotoxic T lymphocyte (CTL) avidity in high dose antigen induction of apoptosis of effector CTL J Exp Med 1996 184 485 492 10.1084/jem.184.2.485 8760802 PMC2192715

[B35] Thoma-Uszynski S Stenger S Modlin R CTL-Mediated Killing of Intracellular Mycobacterium tuberculosis Is Independent of Target Cell Nuclear Apoptosis J Immunol 2000 165 5773 5779 11067936 10.4049/jimmunol.165.10.5773

[B36] Yu T Yang YH Dong DQ The role of CD8+CD28-regulatory T lymphocytes in pulmonary tuberculosis Zhonghua Jie He He Hu Xi Za Zhi 2007 30 130 132 17445477

[B37] Yang Y Tang SJ Zhang Q Sun H Liu YD Hao XH Yao L Gu J The changes and the significance of cellular immune function in peripheral blood of patients with multidrug-resistant and extensively drug-resistant pulmonary tuberculosis Zhonghua Jie He He Hu Xi Za Zhi 2011 34 109 113 21426728

